# Global wealth disparities drive adherence to COVID-safe pathways in head and neck cancer surgery

**DOI:** 10.1093/bjsopen/zrab112

**Published:** 2021-12-27

**Authors:** COVIDSurg Collaborative, Andrew G Schache, Richard Shaw, Michael Wing Sung Ho, Stuart C Winter, James Glasbey, Ian Ganly, Martin Batstone, Juan Rey Biel, Paul C Nankivell, Christian Simon, Omar Omar, Joana F F Simoes, Dmitri Nepogodiev, Aneel Bhangu, Tom Pinkney, Laura McGill, Rita Perry, Terry Hughes, Richard Jackson, Kwabena Siaw-Acheampong, Ruth A Benson, Edward Bywater, Daoud Chaudhry, Brett E Dawson, Jonathan P Evans, James C Glasbey, Rohan R Gujjuri, Emily Heritage, Conor S Jones, Sivesh K Kamarajah, Chetan Khatri, Rachel A Khaw, James M Keatley, Andrew Knight, Samuel Lawday, Elizabeth Li, Harvinder S Mann, Ella J Marson, Kenneth A McLean, Siobhan C Mckay, Emily C Mills, Dmitri Nepogodiev, Gianluca Pellino, Maria Picciochi, Elliott H Taylor, Abhinav Tiwari, Joana F F Simoes, Isobel M Trout, Mary L Venn, Richard J W Wilkin, Aneel Bhangu, James C Glasbey, Richard Shaw, Andrew G Schache, Stuart C Winter, Michael W S Ho, Paul Nankivell, Juan Rey Biel, Martin Batstone, Ian Ganly, Christian Simon, Joana F F Simoes, Tom E F Abbott, Michel Adamina, Adesoji O Ademuyiwa, Arnav Agarwal, Ehab Alameer, Derek Alderson, Felix Alakaloko, Markus Albertsmeiers, Osaid Alser, Muhammad Alshaar, Sattar Alshryda, Alexis P Arnaud, Knut Magne Augestad, Faris Ayasra, José Azevedo, Brittany K Bankhead-Kendall, Emma Barlow, Ruth A Benson, Ruth Blanco-Colino, Amanpreet Brar, Ana Minaya-Bravo, Kerry A Breen, Chris Bretherton, Igor Lima Buarque, Joshua Burke, Edward J Caruana, Mohammad Chaar, Sohini Chakrabortee, Peter Christensen, Daniel Cox, Moises Cukier, Miguel F Cunha, Giana H Davidson, Anant Desai, Salomone Di Saverio, Thomas M Drake, John G Edwards, Muhammed Elhadi, Sameh Emile, Shebani Farik, Marco Fiore, J Edward Fitzgerald, Samuel Ford, Tatiana Garmanova, Gaetano Gallo, Dhruv Ghosh, Gustavo Mendonça Ataíde Gomes, Gustavo Grecinos, Ewen A Griffiths, Madalegna Gründl, Constantine Halkias, Ewen M Harrison, Intisar Hisham, Peter J Hutchinson, Shelley Hwang, Arda Isik, Michael D Jenkinson, Pascal Jonker, Haytham M A Kaafarani, Angelos Kolias, Schelto Kruijff, Ismail Lawani, Hans Lederhuber, Sezai Leventoglu, Andrey Litvin, Andrew Loehrer, Markus W Löffler, Maria Aguilera Lorena, Maria Marta Madolo, Piotr Major, Janet Martin, Hassan N Mashbari, Dennis Mazingi, Symeon Metallidis, Ana Minaya-Bravo, Helen M Mohan, Rachel Moore, David Moszkowicz, Susan Moug, Joshua S Ng-Kamstra, Mayaba Maimbo, Milagros Niquen, Faustin Ntirenganya, Maricarmen Olivos, Kacimi Oussama, Oumaima Outani, Marie Dione Parreno-Sacdalanm, Francesco Pata, Carlos Jose Perez Rivera, Thomas D Pinkney, Willemijn van der Plas, Peter Pockney, Ahmad Qureshi, Dejan Radenkovic, Antonio Ramos-De la Medina, Keith Roberts, April C Roslani, Martin Rutegård, Irène Santos, Sohei Satoi, Raza Sayyed, Andrew Schache, Andreas A Schnitzbauer, Justina O Seyi-Olajide, Neil Sharma, Richard Shaw, Sebastian Shu, Kjetil Soreide, Antonino Spinelli, Grant D Stewart, Malin Sund, Sudha Sundar, Stephen Tabiri, Philip Townend, Georgios Tsoulfas, Gabrielle H van Ramshorst, Raghavan Vidya, Dale Vimalachandran, Oliver J Warren, Duane Wedderburn, Naomi Wright, L A Boccalatte, M Batstone, R Hodge, J Abeloos, T De Backer, J De Ceulaer, C Dick, A Diez-Fraile, P Lamoral, C Spaas, D L A L Schrijvers, E B M Willemse, C Faris, S Maariën, G Van Haesendonck, C Van Laer, P Deron, E A Abdallah, G B Carvalho, L Kowalski, J Vartanian, A P Gatti, C N Nardi, R N L Oliva, M C Salem, D Cheng, D MacNeil, J Martin, R Mayer, G Groot, L Acosta, M Mejia, C J Perez, M Lorencin, I Luksic, M Mamic, F M Ashoush, N A Osman, M Safwat Shahine, A Eldaly, M M A Elfiky, A Amin, R Elmorsi, B Refky, M M Essa, G Mengesha Mengistu, S Dakpé, S Boucher, Q Ballouhey, J Laloze, J Usseglio, C Hoffmann, V Gregoire, B Lallemant, M Blaurock, D Reim, A Boehm, O Guntinas-Lichius, F Hölzle, A Modabber, P Winnand, J Kleeff, K Lorenz, U Ronellenfitsch, R Schneider, C S Betz, A Böttcher, C Busch, N Möckelmann, J M Inhestern, J Greve, T K Hoffmann, S Laban, J M Vahl, A Agyeman-Prempeh, D Aning, I Barnor, R Darko-Asante, M Dzogbefia, V Gaveh, D Gyimah, M D Issahalq, A Konney, M Poku, T Adjeso, E T Akornor, W O Amankwaa, D A Antwi, P Apppiah-Thompson, M Damah, E O Kumi, L Manan, J P Murphy, L Osei, J Setuagbe, N Arkadopoulos, N Danias, P Economopoulou, M Frountzas, P Kokoropoulos, A Larentzakis, N V Michalopoulos, K Nastos, S Parasyris, E Pikoulis, J Selmani, T A Sidiropoulos, P Vassiliu, C E Kalfountzos, I Chatziioannou, C Corais, E Gkrinia, A Ntziovara, A Saratziotis, K Antoniadis, O Orestis, D Tatsis, E Baili, A Charalabopoulos, T Liakakos, D Schizas, E Spartalis, A Syllaios, C Zografos, M Aguilera-Arévalo, S Misra, P Pareek, J Vishnoi, P Chappity, M Kar, D K Muduly, M Sultania, S Agarwal, P K Garg, D D Maharaj, K S Majumdar, N Mishra, D Poonia, R K Seenivasagam, M P Singh, A R Tiwari, P Penumadu, S Rajan, S Kumar, R Raychowdhury, R Ghodke, R Raychowdhury, C Barry, D Callanan, A Dias, L Haung, A Ionescu, P Sheahan, P Lennon, C Fitzgerald, A Mizrachi, A Deganello, R Pellini, B Pichi, F Lemma, M V Marino, M Bergonzani, A Varazzani, F Bussu, T Perra, A Piras, A Porcu, D Rizzo, G Campisi, A Cordova, M Franza, G Rinaldi, F Toia, A Gianni, L Giannini, L Gordini, E Baldini, L Conti, A De Virgilio, F Ferreli, F Gaino, G Mercante, G Spriano, M Ansarin, F Chu, R De Berardinis, G Ietrobon, M Tagliabue, F Ionna, A Baietti, P Maremonti, F Neri, G Prucher, S Ricci, A Casaril, M Nama, A Cotoia, V Lizzi, F Vovola, P Bruzzaniti, P Familiari, P Lapolla, G Marruzzo, A Mingoli, D Ribuffo, R Cipriani, F Contedini, M Lauretta, C Marchetti, M Melotti, M Pignatti, V Pinto, A Pizzigallo, F Ricotta, A Tarsitano, L Catarzi, G Consorti, E A Abdulwahed, E A Alshareea, M I Biala, R J Ghmagh, A F Ibrahim, Y T Liew, M R Alvarez, R Arrangoiz, F Cordera, A Gómez-Pedraza, C E Soulé-Martínez, O S Becerril, GFC Becerra, Y Arkha, H Bechri, A El Ouahabi, M Oudrhiri, A Benkabbou, M Majbar, R Mohsine, A Souadka, N Lageju, W H Schreuder, J Hardillo, R de Bree, D Schweitzer, A A Adeyeye, E E Enoch, T S T T Sholadoye, F Wuraola, O Oyelakin, M I Khokhar, B Ayub, M Walędziak, M Szewczyk, C Faria, P Cardoso, J Castro Silva, E AlKharashi, D Jelovac, M Petrovic, S Sumrak, R R Asceric, J M Bojicic, B M Kovacevic, I D Krdzic, M A Milentijevic, V Z Milutinovic, Z B Stefanovic, J M Villacampa, V Jiménez Carneros, A Salazar Carrasco, A Carabias Hernandez, L Alonso Lamberti, R León Ledesma, F J Jiménez Miramón, J M Jover Navalón, J Garcia Quijada, J Ramos Rodriguez, A Valle Rubio, I Vilaseca, J Escartin, M Estaire-Gomez, D Padilla Valverde, M Tousidonis, F Lopez, U Deandrés-Olabarria, M Durán-Ballesteros, F Fernández-Pablos, F Ibáñez-Aguirre, A Sanz-Larrainzar, B Ugarte-Sierra, M Di Martino, J Prada, U M Jariod-Ferrer, A Landaluce Olavarria, J Rey-Biel, P Díaz de Cerio, A Sánchez Barrueco, E K Lindqvist, M Sund, R Piantanida, R Giger, S Hool, S A Müller, S J Stoeckli, C Simon, T Toutounji, A Al assaf, A M Hammed, S M Hammed, M Mahfoud, A Arikan, Ö Yalkin, N İflazoğlu, A Isik, S Leventoglu, L Aydemir, B Basaran, C Sen, M Comert -Ulusan, B Basaran, K T Saracoglu, A Saracoglu, B Mantoglu, G Kucuk, N Aygun, E Baran, M Tanal, A Eray Tufan, M Uludag, S Gürkan Yetkin, B Yigit, B Calik, S Demirli Atici, T Kaya, F K Sikakulya, K M A H Abdel-Galil, T Lowe, A J Durrani, A Habeeb, E Irune, L Luke, L Masterson, S H Murphy, N Segaren, C Walker, S Waseem, T M Jones, C Loh, S Pringle, A G Schache, R J Shaw, J Stenhouse, M Armstrong, S Sood, D Sutton, S Thomas, P Clarke, S C Winter, S Hislop, P R Counter, N Ghazali, C Lloyd, V Prabhu, D Godden, S Whitley, C Butler, R Nash, K El-Boghdadly, A Fry, R Niziol, M De, C K Gill, S Crank, A D Mace, M Ho, M Mair, P Kothari, J Homer, S Sainuddin, R J Egan, M Kittur, C Burgess, J O'Hara, J Manickavasagam, C McDonald, S Burrows, K R Java, C Katre, A Ahmed, H Siddique, E King, P Ramchandani, P R Naredla, P Brennan, T Ringrose, F Schmidt, J K C Mak, P Nankivell, S Parmar, N Sharma, C Douglas, J McCaul, J McCaul, J Dhanda, N Ghazali, P Kyzas, L Vassiliou, A Kumar, A Husband, J Hulbert, D Ingrams, R Parkin, I Varley, D Gahir, A George, D Zakai, M Bater, C Surwald, B Devlin, C G Leonard, N Pigadas, D Snee, R P Singh, N C Hyde, M Paley, H Cocks, A Wilson, D Choi, C J Kerawala, F Riva, A Dickason, C J Semple, C Schilling, P R Naredla, G Walton, O Rees-Stoner, N Scott, I J Nixon, D Tighe, S Mattine, M M H Chu, V Pothula, W Lee, L Brown, I Ganly, N Alpert, C N Illezeau, B Miles, J Rapp, E Taioli, M T Azam, A J Choudhry, W Marx, J Stein, Y Ying, N D Gross, M Almasri, R Joshi, G Kulkarni, H Marwan, M Mehdi, B Sumer

*Dear Editor*

The COVID-19 pandemic has had profound impacts on safe healthcare delivery, particularly within surgical specialties^1^. There has been clear evidence of pulmonary complications and death associated with surgery following perioperative COVID infection^2^^,^^3^. Mitigation of COVID-related risk for patients depends upon several primary tenets of safe surgery, including implementation of appropriate COVID-secure treatment pathways^4^, provision of adequate testing for patients to ensure COVID-negative status and protection of staff members through suitable personal protective equipment (PPE). Following the initial peaks of SARS-CoV-2 infection, healthcare service provision was afforded time for preparation and reorganization before subsequent waves of infection. An ability to use this window of opportunity was dependent not only on strategic decision making but also national financial resources and healthcare capabilities.

Vaccines against the SARS-CoV-2 virus have demonstrated efficacy, however timely accessibility for healthcare workers, let alone the global population, remains inequitable^5^. The prioritization of healthcare workers alongside surgical patients is essential to impact perioperative mortality. Despite international roll-outs of vaccination, repeated waves of COVID-19 infection are apparent across all continents.

Utilizing an international survey of practice, distributed through an existing COVIDSurg head and neck (HN) collaborative, social media and international HN clinical networks, the authors sought to assess the extent to which COVID-safe HN cancer surgery and individual surgeon-specific protection were implemented between the initial stages of the pandemic (March–June 2020) and a subsequent period in February 2021 after well documented increases in COVID-19 incidence globally and an evolving, evidence-based understanding of the disease (*Supplementary material*).

Analysis of 213 responses from HN institutions situated in 39 countries (*Table S1*), demonstrated a clear disparity in the implementation of patient and staff COVID testing, in PPE usage, and in surgeon-level vaccination when classified by national wealth (World Bank national income classification) (*Table 1*).

**Table 1:** Change in COVID-19 testing, personal protective equipment usage and COVID vaccination during the pandemic

Measure of COVID-safe surgical pathway		**Percentage using preoperative COVID-19 testing** †				**Percentage of centres with routine staff testing**‡			
	n*	Mar–Jun 2020	Feb 2021	Difference	*P*	Mar–Jun 2020	Feb 2021	Difference	*P*
**Global response**									
All centres	213 (100)	76.3 (71.6, 81.0)	88.6 (84.6, 92.6)	12.4 (7.4, 17.4)	<0.001¶	13.6 (9.9, 17.3)	29.4 (23.9, 34.9)	15.8 (10.2, 21.4)	<0.001¶
**World Bank national income group**									
High	156 (73)	78.1 (72.8, 83.4)	94.8 (91.6, 98.0)	16.8 (11, 22.6)		12.9 (8.7, 17.1)	37.8 (31, 44.6)	24.9 (18.2, 31.6)	
Upper middle	27 (13)	84 (73.1, 94.9)	81.3 (67.2, 95.4)	−2.7 (−17.1, 11.7)		26.9 (11.8, 42)	11.2 (0.9, 21.5)	−15.8 (−28.4, 3.2)	
Lower middle	26 (12)	63.2 (47.6, 78.8)	66.5 (50.2, 82.8)	3.3 (−10.3, 16.9)		6.3 (0, 13.2)	3.1 (−2.4, 8.6)	−3.1 (−7.1, 0.9)	
Low	4 (2)	43.8 (−0.3–87.9)	43.8 (−5.7–93.3)	0 (−8–8)		5 (0, 14.8)	0 (0, 0)	−5 (−14.8, 4.8)	
**Comparison**					<0.001#				<0.001#

**Measure of COVID-safe surgical pathway**		**Percentage of surgical procedures with PPE**					**Estimated percentage of staff vaccinated§**		
	n*	**Mar–Jun 2020**	**Feb 2021**	**Difference**	***P***	**Feb 2021**			***P***

**Global response**									
All centres	213 (100)	68.4 (63.2, 73.6)	62.2 (56.6, 67.8)	−6.2 (−10.2, 2.2)	0.120¶	68.4 (63.2, 73.6)			<0.001¶
**World Bank national income group**									
High	156 (73)	66.5 (60.3, 72.7)	62.1 (55.6, 68.6)	−4.4 (−9.1, 0.3)		79.4 (74.3, 84.5)			
Upper middle	27 (13)	80.8 (68.6, 93)	72.3 (58.0, 86.6)	−8.5 (−18.7, 1.7)		64.2 (45.9, 82.5)			
Lower middle	26 (12)	74 (60.2, 87.8)	58.9 (41.8, 76.0)	−15.1 (−26.6, 3.6)		37.2 (22.0, 52.4)			
Low	4 (2)	25 (−24.0, 74.0)	25 (−24.0, 74.0)	0 (0, 0)		0 (0, 0)			
**Comparison**					0.005#				0.001#

Values in parentheses are 95 per cent confidence intervals unless indicated otherwise;

* values in parentheses are percentages.

† Within 72 hours of surgery.

‡ Testing at least weekly.

§ At least one dose of SARS-CoV-2 vaccine.

¶ *P* values test differences between March–June 2020 and Feb 2021.

# *P* values test whether variance in estimates (between March–June 2020 and Feb 2021) is consistent between income groups. *P* ≤ 0.050 indicates significant difference by income group. PPE, personal protective equipment.

Despite evidence of consistent international improvements since the early stages of the pandemic, by February 2021, centres in low- and low–middle-income countries were still significantly less likely to employ routine preoperative COVID testing (60 per cent) or PPE use (49 per cent) compared with global averages (89 and 62 per cent respectively).

Further evidence of significant global inequality was apparent in disparate vaccination access: less than one third of surgical staff in low- and low–middle-income countries received a first vaccine dose by spring 2021 (international average 68.4 (95 per cent c.i. 63.2 to 73.6) per cent).

HN centres in countries experiencing highest levels of population COVID incidence (25 or more per 100 000 population) in spring 2021 correlated to highest levels of testing, PPE use and vaccination (*Table S2*). However, there was an international trend towards reduction in PPE use over time, particularly pronounced in UK centres, and within hospitals designated as cold sites (*Table S3*). Although ongoing fluctuations in PPE availability may have influenced this pattern, growing ambivalence or over-reliance on testing and/or vaccination may also have contributed, such that the role of PPE in HN surgery is deemed less relevant by surgeons.

Reported COVID-19 infections amongst surgeons (16 per cent) within this survey are at least double those of conservative contemporaneous population-level estimates. In countries yet to receive nationwide vaccination, HN surgeons had even higher levels of prior COVID infection (26 per cent), highlighting further global wealth inequalities and populations with potentially greater latent need.

Whilst the authors recognize inherent limitations to these data, including the intrinsic bias associated with surveys, it is evident that global wealth inequality appears to drive disparities in access to critical elements of appropriate healthcare, risking both patient and staff safety.

HN surgery exemplifies many of the highest risks of surgery in general and remains critically reliant on COVID-secure pathways supported by robust testing, adequate PPE provision and vaccination. These data share yet another indicator of inequitable distribution of healthcare resource at a time when we are all reliant upon worldwide solutions.

## Collaborators

***Writing group***

Andrew G Schache, Richard Shaw, Michael Wing Sung Ho, Stuart C Winter, James Glasbey, Ian Ganly, Martin Batstone, Juan Rey Biel, Paul C Nankivell, Christian Simon, Omar Omar, Joana FF Simoes, Dmitri Nepogodiev, Aneel Bhangu, Tom Pinkney, Laura McGill, Rita Perry, Terry Hughes, Richard Jackson.

***CovidSurg Operations Committee***

Kwabena Siaw-Acheampong, Ruth A Benson, Edward Bywater, Daoud Chaudhry, Brett E Dawson, Jonathan P Evans, James C Glasbey, Rohan R Gujjuri, Emily Heritage, Conor S Jones, Sivesh K Kamarajah, Chetan Khatri, Rachel A Khaw, James M Keatley, Andrew Knight, Samuel Lawday, Elizabeth Li, Harvinder S Mann, Ella J Marson, Kenneth A McLean, Siobhan C Mckay, Emily C Mills, Dmitri Nepogodiev, Gianluca Pellino, Maria Picciochi, Elliott H Taylor, Abhinav Tiwari, Joana FF Simoes, Isobel M Trout, Mary L Venn, Richard JW Wilkin, Aneel Bhangu.

International Cancer Leads (*denotes specialty Principal Investigator)

James C Glasbey (*Chair*); Head and neck: Richard Shaw*, Andrew G Schache, Stuart C Winter, Michael W S Ho, Paul Nankivell, Juan Rey Biel, Martin Batstone, Ian Ganly, Christian Simon.

***Dissemination Committee***

Joana FF Simoes (*Chair*); Tom EF Abbott, Michel Adamina, Adesoji O Ademuyiwa, Arnav Agarwal, Ehab Alameer, Derek Alderson, Felix Alakaloko, Markus Albertsmeiers, Osaid Alser, Muhammad Alshaar, Sattar Alshryda, Alexis P Arnaud, Knut Magne Augestad, Faris Ayasra, José Azevedo, Brittany K Bankhead-Kendall, Emma Barlow, Ruth A Benson, Ruth Blanco-Colino, Amanpreet Brar, Ana Minaya-Bravo, Kerry A Breen, Chris Bretherton, Igor Lima Buarque, Joshua Burke, Edward J Caruana, Mohammad Chaar, Sohini Chakrabortee, Peter Christensen, Daniel Cox, Moises Cukier, Miguel F Cunha, Giana H Davidson, Anant Desai, Salomone Di Saverio, Thomas M Drake, John G Edwards, Muhammed Elhadi, Sameh Emile, Shebani Farik, Marco Fiore, J Edward Fitzgerald, Samuel Ford, Tatiana Garmanova, Gaetano Gallo, Dhruv Ghosh, Gustavo Mendonça Ataíde Gomes, Gustavo Grecinos, Ewen A Griffiths, Madalegna Gründl, Constantine Halkias, Ewen M Harrison, Intisar Hisham, Peter J Hutchinson, Shelley Hwang, Arda Isik, Michael D Jenkinson, Pascal Jonker, Haytham MA Kaafarani, Angelos Kolias, Schelto Kruijff, Ismail Lawani, Hans Lederhuber, Sezai Leventoglu, Andrey Litvin, Andrew Loehrer, Markus W Löffler, Maria Aguilera Lorena, Maria Marta Madolo, Piotr Major, Janet Martin, Hassan N Mashbari, Dennis Mazingi, Symeon Metallidis, Ana Minaya-Bravo, Helen M Mohan, Rachel Moore, David Moszkowicz, Susan Moug, Joshua S Ng-Kamstra, Mayaba Maimbo, Milagros Niquen, Faustin Ntirenganya, Maricarmen Olivos, Kacimi Oussama, Oumaima Outani, Marie Dione Parreno-Sacdalanm, Francesco Pata, Carlos Jose Perez Rivera, Thomas D Pinkney, Willemijn van der Plas, Peter Pockney, Ahmad Qureshi, Dejan Radenkovic, Antonio Ramos-De la Medina, Keith Roberts, April C Roslani, Martin Rutegård, Irène Santos, Sohei Satoi, Raza Sayyed, Andrew Schache, Andreas A Schnitzbauer, Justina O. Seyi-Olajide, Neil Sharma, Richard Shaw, Sebastian Shu, Kjetil Soreide, Antonino Spinelli, Grant D Stewart, Malin Sund, Sudha Sundar, Stephen Tabiri, Philip Townend, Georgios Tsoulfas, Gabrielle H van Ramshorst, Raghavan Vidya, Dale Vimalachandran, Oliver J Warren, Duane Wedderburn, Naomi Wright, EuroSurg, European Society of Coloproctology (ESCP), Global Initiative for Children’s Surgery (GICS), GlobalSurg, GlobalPaedSurg, ItSURG, PTSurg, SpainSurg, Italian Society of Colorectal Surgery (SICCR), Association of Surgeons in Training (ASiT), Irish Surgical Research Collaborative (ISRC), Transatlantic Australasian Retroperitoneal Sarcoma Working Group (TARPSWG), Italian Society of Surgical Oncology (SICO).

***Collaborating authors***

**Argentina**: Boccalatte LA (*Hospital Italiano de Buenos Aires*).

**Australia:** Batstone M, Hodge R (*Royal Brisbane and Women's Hospital*).

**Belgium**: Abeloos J, De Backer T, De Ceulaer J, Dick C, Diez-Fraile A, Lamoral P, Spaas C (*AZ Sint-Jan Brugge-Oostende AV*); Schrijvers DLAL (*Hospital Network Antwerp*); Willemse EBM (*Jules Bordet Institute*); Faris C, Maariën S, Van Haesendonck G, Van Laer C (*University Hospital Antwerp);* Deron P (*University Hospital Gent*).

**Brazil**: Abdallah EA, Carvalho GB, Kowalski L, Vartanian J (*A.C. Camargo Cancer Center*); Gatti AP, Nardi CN, Oliva RNL (*Hospital Geral de Pirajussara*); Salem MC (*Irmandade da Santa Casa de Misericórdia de Porto Alegre*).

**Canada**: Cheng D, MacNeil D, Martin J, Mayer R, (*London Health Sciences Centre and St Josephs Health Care London*); Groot G (*Saskatoon City Hospital/Royal University Hospital/St. Paul's Hospital*).

**Colombia**: Acosta L, Mejia M, Perez CJ (*Fundacion Cardioinfantil-IC*).

**Croatia**: Lorencin M, Luksic I, Mamic M (*University Hospital Dubrava*).

**Egypt**: Ashoush FM (*Alexandria Main University Hospital*); Osman NA (*Alexandria Medical Research Institute*); Safwat Shahine M (*Assiut University Hospital*); Eldaly A (*El-Menshawy Hospital*); Elfiky MMA (*Kasr Alainy Hospital*); Amin A (*National Cancer Institute*); Elmorsi R, Refky B (*Oncology Center Mansoura University*); Essa MM (*Tanta University Hospital*).

**Ethiopia**: Mengistu G Mengesha (*Hawassa University Comprehensive Specialized Hospital*).

**France**: Dakpé s (*CHU Amiens*); Boucher S (*CHU Angers*); Ballouhey Q, Laloze J, Usseglio J (*CHU Limoges*); Hoffmann C (*Curie Institute*); Gregoire V (*Léon Bérard Cancer Center*); Lallemant B (*University Hospital of Nîmes*).

**Germany**: Blaurock M (*Greifswald University Medicine*); Reim D (*Klinikum Rechts der Isar TUM School of Medicine*); Boehm A (*Klinikum St. Georg, ENT Dept.*); Guntinas-Lichius O (*Universitätsklinikum Jena*); Hölzle F, Modabber A, Winnand P (*University Hospital Aachen*); Kleeff J, Lorenz K, Ronellenfitsch U, Schneider R (*University Hospital Halle, Saale*); Betz CS, Böttcher A, Busch C, Möckelmann N (*University Medical Center Hamburg-Eppendorf*); Inhestern JM (*Oberhavelkliniken Hennigsdorf*) Greve J, Hoffmann TK, Laban S, Vahl JM (*University Hospital Ulm*).

**Ghana**: Agyeman-Prempeh A, Aning D, Barnor I, Darko-Asante R, Dzogbefia M, Gaveh V, Gyimah D, Issahalq MD, Konney A, Poku M (*Komfo-Anokye Teaching Hospital*); Adjeso T, Akornor ET, Amankwaa WO, Antwi DA, Apppiah-Thompson P, Damah M, Kumi EO, Manan L, Murphy JP, Osei L, Setuagbe J (*Tamale Teaching Hospital*).

**Greece**: Arkadopoulos N, Danias N, Economopoulou P, Frountzas M, Kokoropoulos P, Larentzakis A, Michalopoulos NV, Nastos K, Parasyris S, Pikoulis E, Selmani J, Sidiropoulos TA, Vassiliu P (*Attikon University General Hospital*); Kalfountzos CE (*General Hospital of Larissa "Koutlimpaneio and Triantafylleio"*); Chatziioannou I, Corais C, Gkrinia E, Ntziovara A, Saratziotis A (*General University Hospital of Larissa*); Antoniadis K, Orestis O, Tatsis D (*George Papanikolaou General Hospital of Thessaloniki*); Baili E, Charalabopoulos A, Liakakos T, Schizas D, Spartalis E, Syllaios A, Zografos C (*Laiko University Hospital*).

**Guatemala**: Aguilera-Arévalo M (*Hospital General San Juan De Dios*).

**India**: Misra S, Pareek P, Vishnoi J (*All India Institute of Medical Sciences, AIIMS, Jodhpur*); Chappity P, Kar M, Muduly DK, Sultania M (*All India Institute Of Medical Sciences, Bhubhaneshwar*); Agarwal S, Garg PK, Maharaj DD, Majumdar KS, Mishra N, Poonia D, Seenivasagam RK, Singh MP, Tiwari AR (*All India Institute Of Medical Sciences, Rishikesh*); Penumadu P (*Jawaharlal Institute of Postgraduate Medical Education and Research*); Rajan S (*King George's Medical University, Lucknow, UP*); Kumar S (*Max Superspeciality Hospital*); Raychowdhury R (*Ramakrishna Mission Seva Pratishthan*); Ghodke R (*Sanjay Clinic & Apollo Hospital*); Raychowdhury R (*Vivekananda Institute of Medical Sciences*).

**Ireland**: Barry C, Callanan D, Dias A, Haung L, Ionescu A, Sheahan P (*South Infirmary Victoria University Hospital*); Lennon P, Fitzgerald C (*St. James's Hospital*).

**Israel:** Mizrachi A (*Rabin Medical Center*).

**Italy**: Deganello A (*ASST Spedali Civili, Ospedale di Brescia*); Pellini R, Pichi B (*Regina Elena National Cancer Institute*); Lemma F (*Azienda Ospedaliera di Padova*); Marino MV (*Azienda Ospedaliera, Ospedali Riuniti Villa Sofia, Cervello*); Bergonzani M, Varazzani A (*Azienda Ospedaliero-Universitaria di Parma*); Bussu F, Perra T, Piras A, Porcu A, Rizzo D (*Cliniche San Pietro, A.O.U. Sassari*); Campisi G, Cordova A, Franza M, Rinaldi G, Toia F (*Department of Surgical, Oncological and Oral Sciences, University of Palermo*); Gianni A (*Fondazione IRCCS Ca' Granda - ospedale Maggiore Policlinico*); Giannini L (*Fondazione IRCCS Istituto Nazionale dei Tumori, Milano*); Gordini L (*Fondazione Policlinico Universitario Agostino Gemelli*); Baldini E, Conti L (*G. Da Saliceto*); De Virgilio A, Ferreli F, Gaino F, Mercante G, Spriano G (*Humanitas University; IRCCS Humanitas Research Hospital*); Ansarin M, Chu F, De Berardinis R, Ietrobon G, Tagliabue M (*Istituto Europeo di Oncologia - IRCCS -Milano*); Ionna F (*Istituto Nazionale Tumori Fondazione, Pascale - IRCCS*); Baietti A, Maremonti P, Neri F, Prucher G, Ricci S (*Ospedale Maggiore/Bellaria Carlo Alberto Pizzardi AUSL Bologna*); Casaril A, Nama M (*Ospedale Pederzoli*); Cotoia A, Lizzi V, Vovola F (*Ospedali Riuniti Azienda Ospedaliera Universitaria Foggia*); Bruzzaniti P, Familiari P, Lapolla P, Marruzzo G, Mingoli A, Ribuffo D (*Policlinico Umberto I*); Cipriani R, Contedini F, Lauretta M, Marchetti C, Melotti M, Pignatti M, Pinto V, Pizzigallo A, Ricotta F, Tarsitano A (*Sant'Orsola Hospital, Alma Mater Studiorum University of Bologna, Italy*); Catarzi L, Consorti G (*University Hospital Umberto, Ancona*).

***Libya***: Abdulwahed EA, Alshareea EA, Biala MI, Ghmagh RJ (*Tripoli Central Hospital*).

**Malaysia**: Ibrahim AF (*Sarawak General Hospital*); Liew YT (*University Malaya Medical Centre*).

**Mexico**: Alvarez MR, Arrangoiz R, Cordera F, Gómez-Pedraza A (*ABC Medical Center*); Soulé-Martínez CE (*Hospital Central Norte Pemex*); Becerril OS (*Hospital Juárez de México*); Becerra GFC (*Hospital San Angel Inn Patriotismo*).

**Morocco**: Arkha Y, Bechri H, El Ouahabi A, Oudrhiri M (*Hôpital des Spécialités - ONO*); Benkabbou A, Majbar M, Mohsine R, Souadka A (*Universite Mohammed V Institut National d'Oncologie Rabat*).

**Nepal**: Lageju N (*Nepal Police Hospital*).

**Netherlands**: Schreuder WH (*Antoni van Leeuwenhoek Ziekenhuis*); Hardillo J (*Erasmus Medisch Centrum*); de Bree R (*University Medical Center Utrecht*); Schweitzer D (*Zuyderland Medical Center*).

**Nigeria**: Adeyeye AA, Enoch EE (*Afe Babalola University Multi-System Hospital*); Sholadoye TSTT (*Ahmadu Bello University Teaching Hospital*); Wuraola F (*Obafemi Awolowo University Teaching Hospitals Complex*); Oyelakin O (*University College Hospital, Ibadan*).

**Pakistan**: Khokhar MI (*Lahore General Hospital, PGMI, AMC*); Ayub B (*Patel Hospital*).

**Poland**: Walędziak M (*Military Institute Of Medicine*); Szewczyk M (*The Gerater Poland Cancer Center*).

**Portugal**: Faria C (*Centro Hospitalar Sao Joao*); Cardoso P (*Hospital Faro, Centro Hospitalar Universitario do Algarve*); Castro Silva J (*IPO Porto*).

**Saudi Arabia**: AlKharashi E (*Prince sultan military medical city*).

**Serbia**: Jelovac D, Petrovic M, Sumrak S (*Clinic for Maxillofacial Surgery, School of Dental Medicine, University of Belgrade*); Asceric RR, Bojicic JM, Kovacevic BM, Krdzic ID, Milentijevic MA, Milutinovic VZ, Stefanovic ZB (*Zvezdara University Medical Center*).

**Spain**: Villacampa JM (*Fundación Jimenez Diaz University Hospital*); Jiménez Carneros V, Salazar Carrasco A, Carabias Hernandez A, Alonso Lamberti L, León Ledesma R, Jiménez Miramón FJ, Jover Navalón JM, Garcia Quijada J, Ramos Rodriguez J, Valle Rubio A (*Getafe University Hospital*); Vilaseca I (*Hospital Clínic of Barcelona*); Escartin J (*Hospital Royo Villanova*); Estaire-Gomez M, Padilla Valverde D (*Hospital General Universitario de Ciudad Real*); Tousidonis M (*Hospital General Universitario Gregorio Marañón*); Lopez F (*Hospital Universitario Central de Asturias, HUCA*); Deandrés-Olabarria U, Durán-Ballesteros M, Fernández-Pablos F, Ibáñez-Aguirre F, Sanz-Larrainzar A, Ugarte-Sierra B (*Hospital Universitario de Galdakao*); Di Martino M, Prada J (*Hospital Universitario de la Princesa*); Jariod-Ferrer UM (*Hospital Universitario Miguel Servet*); Landaluce Olavarria A (*Hospital Urduliz*); Rey-Biel J (*Rey Juan Carlos University Hospital*); Díaz de Cerio P (*Saint Peter Hospital*); Sánchez Barrueco A (*Villalba University General Hospital*).

**Sweden**: Lindqvist EK (*Karolinska University Hospital*); Sund M (*Umea University Hospital*).

**Switzerland**: Piantanida R (*Ente Ospedaliero Cantonale*); Giger R, Hool S, Müller SA (*Inselspital, Bern University Hospital, University of Bern*); Stoeckli SJ (*Kantonsspital St. Gallen*); Simon C (*Lausanne University Hospital CHUV*).

**Syrian Arab Republic**: Toutounji T (*Aleppo University Hospital*);Al assaf A (*Al-Mouwasat University Hospital*); Hammed AM, Hammed SM, Mahfoud M (*Tishreen University Hospital*).

**Turkey**: Arikan A (*Acibadem Maslak Hospital*); Yalkin Ö (*Ali Osman Sönmez Oncology Hospital*); İflazoğlu N (*Bursa City Hospital*); Isik A, Leventoglu S (*Erzincan University Hospital*); Aydemir L, Basaran B, Sen C, Comert -Ulusan M (*Istanbul University Faculty of Medicine*); Basaran B (*Karaman Training and Research Hospital*); Saracoglu KT (*Kartal Dr. Lutfi Kirdar Training and Research Hospital*); Saracoglu A (*Marmara University, School of Medicine*); Mantoglu B (*Sakarya Faculty Of Medicine*); Kucuk G (*Samsun Training and Research Hospital*); Aygun N, Baran E, Tanal M, Eray Tufan A, Uludag M, Gürkan Yetkin S, Yigit B (*Sisli Hamidiye Etfal Training and Research Hospital*); Calik B, Demirli Atici S, Kaya T (*University of Health Sciences Tepecik Training and Research Hospital*).

**Uganda**: Sikakulya FK (*Kampala International University Teaching Hospital*).

**United Arab Emirates**: Abdel-Galil KMAH (*Tawam Johns Hopkins Hospital*).

**UK**: Lowe T (*Aberdeen Royal Infirmary*); Durrani AJ, Habeeb A, Irune E, Luke L, Masterson L, Murphy SH, Segaren N, Walker C, Waseem S (*Addenbrooke's Hospital*); Jones TM, Loh C, Pringle S, Schache AG, Shaw RJ (*Aintree University Hospital*); Stenhouse J (*Altnagelvin Area Hospital*); Armstrong M (*Borders General Hospital*); Sood S, Sutton D (*Bradford Royal Infirmary*); Thomas S (*Bristol Royal Infirmary*); Clarke P (*Charing Cross Hospital*); Winter SC (*Churchill Hospital*); Hislop S (*Crosshouse University Hospital*); Counter PR (*Cumberland Infirmary*); Ghazali N (*East Lancashire Hospitals NHS Trust*); Lloyd C (*Glan Clwyd Hospital*); Prabhu V (*Glangwili General Hospital*); Godden D, Whitley S (*Gloucestershire Royal Hospital*); Butler C, Nash R (*Great Ormond Street Hospital for Children*); El-Boghdadly K, Fry A, Niziol R (*Guy's Hospital*); De M, Gill CK (*Heartlands Hospital*); Crank S (*Hull University Teaching Hospitals NHS Trust*); Mace AD (*Imperial College NHS Trust*); Ho M (*Leeds General Infirmary*); Mair M (*Leicester Royal Infirmary*); Kothari P (*Luton and Dunstable University Hospital*); Homer J, Sainuddin S (*Manchester Royal Infirmary*); Egan RJ, Kittur M (*Morriston Hospital Swansea*); Burgess C (*Musgrove Park Hospital)*; O’Hara J (*Newcastle Upon Tyne Hospitals NHS Foundation Trust*); Manickavasagam J, McDonald C (*Ninewells Hospital*); Burrows S (*Norfolk and Norwich University Hospital*); Java KR, Katre C (*North Manchester General Hospital*); Ahmed A, Siddique H (*Northwick Park Hospital*); King E, Ramchandani P (*Poole Hospital*); Naredla PR (*Princess Royal Hospital*) Brennan P, Ringrose T, Schmidt F (*Queen Alexandra Hospital*); Mak JKC, Nankivell P, Parmar S, Sharma N (*Queen Elizabeth Hospital Birmingham*); Douglas C, McCaul J, McCaul J (*Queen Elizabeth University Hospital*); Dhanda J (*Queen Victoria Hospital*); Ghazali N, Kyzas P, Vassiliou L (*Royal Blackburn Hospital*); Kumar A (*Royal Derby Hospital*); Husband A (*Royal Devon and Exeter Hospital*); Hulbert J (*Royal Glamorgan Hospital*); Ingrams D, Parkin R (*Royal Gwent Hospital*); Varley I (*Royal Hallamshire Hospital*); Gahir D, George A, Zakai D (*Royal Stoke University Hospital*); Bater M (*Royal Surrey County Hospital*); Surwald C (*Royal Sussex County Hospital*); Devlin B, Leonard CG (*Royal Victoria Hospital*); Pigadas N, Snee D (*Royal Wolverhampton NHS Trust*); Singh RP (*Southampton General Hospital*); Hyde NC (*St George's Hospital*); Paley M (*St John's hospital, Livingston*); Cocks H, Wilson A (*Sunderland Royal Hospital*); Choi D (*The National Hospital for Neurology and Neurosurgery*); Kerawala CJ, Riva F (*The Royal Marsden NHS Foundation Trust*); Dickason A (*Torbay and South Devon NHS Trust*); Semple CJ (*Ulster Hospital Dundonald*); Schilling C (*University College London Hospital at Westmoreland Street*); Naredla PR, Walton G (*University Hospitals Coventry and Warwickshire NHS Trusts*); Rees-Stoner O (*University Hospitals Plymouth*); Scott N (*University Hospital of Wales*); Nixon IJ (*Western General Hospital*); Tighe D (*William Harvey Hospital*); Mattine S (*Worcestershire Royal Hospital*); Chu MMH, Pothula V (*Wrightington, Wigan & Leigh NHS Foundation Trust*).

**USA**: Lee W (*Duke University Medical Center*); Brown L, Ganly I (*Memorial Sloan Kettering Cancer Center*); Alpert N, Illezeau CN, Miles B, Rapp J, Taioli E (*Mount Sinai Hospital*); Azam MT, Choudhry AJ, Marx W (*SUNY Upstate University Hospital*); Stein J (*The University Hospital*); Ying Y (*University of Alabama Birmingham*); Gross ND (*University of Texas MD Anderson Cancer Center*); Almasri M, Joshi R, Kulkarni G, Marwan H, Mehdi M (*University of Texas Medical Branch*); Sumer B (*UT Southwestern Medical Center*).

## Funding

The research detailed in this manuscript was funded by the British Association of Head & Neck Oncologists (BAHNO).

## Supplementary Material

zrab112_Supplementary_Data
