# Correction to: Global wealth disparities drive adherence to COVID-safe pathways in head and neck cancer surgery

**DOI:** 10.1093/bjsopen/zraf031

**Published:** 2025-03-21

This is a correction to: COVIDSurg Collaborative, Global wealth disparities drive adherence to COVID-safe pathways in head and neck cancer surgery, *BJS Open*, Volume 5, Issue 6, November 2021, https://doi.org/10.1093/bjsopen/zrab112

In the originally published online version of this manuscript, there was an error in a collaborator's name within the body of the article. This should read: “Ioannidis O,” instead of: “Orestis O,”.

This error has been outlined only in this correction notice to preserve the version of record.

