# Supplementary material for: Global wealth disparities drive adherence to COVID-safe pathways in head and neck cancer surgery
Source: BJS Open. 2021 Dec 27;5(6):zrab112. doi: 10.1093/bjsopen/zrab112 (PMC8711291; doi:10.1093/bjsopen/zrab112)
Supplement: zrab112_Supplementary_Data [file zrab112_supplementary_data.docx]

**Methodology - Design**

A REDCap (Research Electronic Data Capture) questionnaire was designed and employed to collate responses and associated data via its web application. A 13-item survey questionnaire was constructed according to previously published guidelines for robust survey design and conduct. In order to ensure optimal question design and to facilitate any necessary refinement, so as to achieve desired respondent interpretation, members of the COVIDSurg-HN writing group undertook both pre-testing and pilot testing evaluation of the questionnaire prior to survey distribution. This process provided assurance of both face and content validity. In the context of the evolving pandemic and in the absence of a gold standard to measure against, no further validity measures were possible.
A subsequent follow up response was requested from all respondents, to clarify both the size of the respective surgical centres (number of patients treated per annum) for which responses were attained and also personal COVID experience of participant surgeons.

**Setting and Participants**

The survey was distributed electronically to the existing COVIDSurg-HN collaborative network; a wide group which had previously contributed to the collection of more than 5000 head and neck cancer treatments carried out between March-June 2020. Additionally, to maximise scope of response, invitations to participate were disseminated both through social media platforms and through the memberships of major international Head & Neck specialty associations and collaborative research/clinical trial networks. Respondents were requested where possible to provide a single response on behalf of their hospital or head and neck cancer multidisciplinary team.

Given the cross-specialty approach to survey dissemination, acquisition of duplicate responses within the dataset was anticipated and, where this occurred, averaging of duplicates was undertaken to provide the best (combined) estimate for any given centre prior to subsequent analysis. Each participating hospital’s data referred to the management of adult patients undergoing head and neck cancer surgery with curative intent. Surgery was defined as a procedure performed by a surgeon in an operating theatre either under general or local anaesthesia. Head and neck cancer was defined as any malignant diagnosis above the clavicle although this excluded localized non-melanoma skin cancer, haematopoietic malignancies, intra-ocular and primary brain malignancy.

**Main Outcome Measures**

The survey data capture was conducted within the first week of February 2021 (1^st^ - 7^th^ February 2021). Other than encouragement of individuals to contribute to a better understanding of the impact of COVID-19 on Head & Neck services, there were no incentives to respond. Completion of the survey by respondents was requested to include estimated metrics of head and neck cancer service. Three distinct timepoints were defined:

- prior to the COVID-19 pandemic (nominally February 2019);
- during the initial stages of COVID-19 infections (March – June 2020);
- at the time of survey completion (1^st^ week of February 2021).

The entry fields represented either percentage estimates, defined response criteria or direct yes/no responses. Responses were classified as required fields for continuation and completion of the survey in all instances. Additional detail of the geographical location of the hospital (country), SARS-CoV-2 testing of patients and staff, usage of Personal Protective Equipment (PPE; FFP3 or equivalent mask usage for H&N operating) and levels of vaccination against COVID-19 was requested from all respondents.

Subsequent to completion and analysis of survey outcomes, all respondents were requested to provide evidence of the size and nature of their respective unit (number of new HN cases treated per annum and COVID pathway designation) and detail of their personal experience of COVID in terms of COVID infection, vaccination and PPE usage.

**Applied Metrics**

International variation in population-level incidence of COVID-19 has been evident since the initial outbreak of disease in late 2019 and throughout the pandemic 2020. The community SARS-CoV-2 incidence within each participating hospitals’ locality was extracted from World Health Organisation statistics. Community SARS-CoV-2 incidence was calculated for the 1^st^ February 2021, the midpoint of the March-June 2020 survey period (15^th^ April) and an average of those 3 months. Hospitals were classified as being in communities with either low (≤25 cases per 100,000 population) or high (>25 cases per 100,000 population) SARS-CoV-2 incidence.

World Bank descriptors of national income and World Health Organisation (WHO) Healthcare system attainment and performance status were applied respectively.

**Statistical Methodology**

Direct comparisons of overall differences between March-June 2020 and February 2021 were performed using paired t-test. Further analyses of data were performed using Analysis of Covariance (ANCOVA) techniques, analysing the February 2021 data whilst including March-June 2020 data. The COVID designation of reporting site (Hot vs Cold; COVID “Hot” sites and those sites without a specific COVID designation yet accepting acute admissions via an Emergency Department (ED) versus COVID “Cold” Sites and those undesignated centres without an ED), Community SARS-CoV-2 incidence (on 1^st^ Feb 2021) and World Bank Income status are also included as covariates.
P-values for the impact of income status are derived from these analyses.

**Income Status of Counties with Responding Head & Neck Centres**

***List of countries from which responses were provided and corresponding World Bank Income Classification groupings and reported population level SARS-CoV-2 infection incidence at specific defined time points. Incidence above 25/100,000 highlighted*****Average community SARS-CoV-2 incidence March-June 2020*

| **Country** | **World Bank Country Income Group** | **COVID-19 Cases/100,000 population March-June 2020*** | **COVID-19 Cases/100,000 population  15th April 2020** | **COVID-19 Cases/100,000 population  1st Feb 2021** |
| --- | --- | --- | --- | --- |
| Argentina | upper middle | 5.02 | 0.23 | 18.44 |
| Australia | high | 0.55 | 0.24 | 0.02 |
| Belgium | high | 4.00 | 12.5 | 20.5 |
| Brazil | upper middle | 9.99 | 0.82 | 33.7 |
| Canada | high | 1.88 | 3.43 | 11.15 |
| Colombia | upper middle | 6.57 | 0.3 | 20.56 |
| Croatia | high | 1.36 | 1.39 | 11.85 |
| Egypt | lower middle | 0.53 | 0.13 | 0.54 |
| Ethiopia | low | 0.25 | 0.004 | 0.53 |
| France | high | 2.67 | 17.8 | 31.77 |
| Germany | high | 1.59 | 3.66 | 12.95 |
| Ghana | lower middle | 0.77 | 0.15 | 2.6 |
| Greece | high | 0.54 | 0.42 | 7.52 |
| Guatemala | upper middle | 2.25 | 0.07 | 3.86 |
| India | lower middle | 1.45 | 0.07 | 0.91 |
| Ireland | high | 3.17 | 18.73 | 24.8 |
| Israel | high | 7.34 | 4.53 | 83.06 |
| Italy | high | 2.41 | 6.08 | 20.01 |
| Libya | upper middle | 1.10 | 0.06 | 10.68 |
| Malaysia | upper middle | 0.16 | 0.42 | 14.21 |
| Mexico | upper middle | 2.53 | 0.3 | 9.44 |
| Morocco | lower middle | 0.92 | 0.29 | 1.85 |
| Nepal | lower middle | 0.74 | 0.003 | 0.59 |
| Netherlands | high | 2.32 | 6.37 | 23.99 |
| Nigeria | lower middle | 0.14 | 0.01 | 0.64 |
| Pakistan | lower middle | 0.73 | 0.16 | 0.64 |
| Poland | high | 0.97 | 0.99 | 14.15 |
| Portugal | high | 3.09 | 6.93 | 109.26 |
| Saudi Arabia | high | 4.93 | 1.2 | 0.75 |
| Serbia | upper middle | 1.95 | 4.53 | 24.56 |
| Spain | high | 5.38 | 8.99 | 67.84 |
| Sweden | high | 4.54 | 4.92 | 27.84 |
| Switzerland | high | 2.65 | 5.04 | 18.56 |
| Syrian Arab Republic (Syria) | low | 0.09 | 0.01 | 0.31 |
| Turkey | upper middle | 1.74 | 5.28 | 8.58 |
| Uganda | low | 0.04 | 0.001 | 16.4 |
| United Arab Emirates | high | 3.86 | 3.9 | 35.39 |
| United Kingdom | high | 2.71 | 6.33 | 34.4 |
| United States of America | high | 9.89 | 8.97 | 42.74 |

**Supplementary Table A**

***Influence of Population level COVID-19 Incidence on metrics of COVID-safe surgery***

|  | **Mar-Jun 2020** (3 month average) | **Feb 2021**  (single day incidence 1/2/2021) | | |
| --- | --- | --- | --- | --- |
|  | **All Hospitals** | **All Hospitals** | **Low COVID Incidence ¥** | **High COVID Incidence ¥** |
| **% receiving pre-op COVID test*** | 76% | 89% | 82% | 96% |
| **% surgical procedures with PPE** | 68% | 62% | 55% | 71% |
| **% centres with routine staff testing†** | 14% | 29% | 15% | 45% |
| **Est. % of staff vaccinated§** | N/A | 71% | 58% | 85% |

| ** within 72 hours of surgery* |
| --- |
| *† testing at least weekly* |
| *§ at least 1 dose of SARS-CoV-2 vaccine* |
| *¥ Low COVID Incidence (≤25/100,000 population)* |
| *High COVID Incidence (>25/100,000 population)* |

**Supplementary Table B**

***COVID-19 Testing & PPE use by individual hospital size and COVID designation.***

|  | **n=**  **(%)** | **% using pre-op COVID-19 testing (range)*** | | **% centres with routine staff testing†** | | **% surgical procedures with PPE** | | **Est. % of staff vaccinated§** |
| --- | --- | --- | --- | --- | --- | --- | --- | --- |
|  |  | **Mar-Jun 2020** | **Feb 2021** | **Mar-Jun 2020** | **Feb 2021** | **Mar-Jun 2020** | **Feb 2021** | **Feb 2021** |
| **Head & Neck Surgical Unit Size (new H&N cases per annum)** | | | | | | | | |
| <100 patients/yr | 49 (28) | 78 | 88 | 18 | 21 | 74 | 70 | 62 |
| 100-399 patients/yr | 94 (53) | 75 | 88 | 14 | 32 | 66 | 61 | 71 |
| 400-799 patients /yr | 21 (12) | 85 | 91 | 13 | 26 | 54 | 38 | 73 |
| >800 patients /yr | 12 (7) | 68 | 91 | 18 | 30 | 65 | 75 | 86 |
| **COVID Designation of Hospital Services** | | | | | | | | |
| Designated COVID “Cold” | 21 (12) | 87% | 88% | 14% | 18% | 61% | 55% | 61% |
| Undesignated without Emergency Department | 8 (5) | 68% | 89% | 9% | 23% | 74% | 53% | 75% |
| Designated COVID “Hot” | 87 (49) | 78% | 88% | 19% | 34% | 62% | 59% | 74% |
| Undesignated with Emergency Department | 60 (34) | 73% | 88% | 13% | 23% | 75% | 70% | 65% |
| *TOTAL* | **176 (100)** | **77 (0-100)** | **88 (0-100)** | **15%** | **28%** | **68 (0-100)** | **62 (0-100)** | **70%** |

| ** within 72 hours of surgery* |
| --- |
| *† testing at least weekly* |
| *§ at least 1 dose of SARS-CoV-2 vaccine* |
| *yr - year* |

**Authorship COVIDSurg Collaborative***

*** Collaborators (PubMed-citable):**

***Writing group († Corresponding Author)***

Andrew G Schache†, Richard Shaw, Michael Wing Sung Ho, Stuart C Winter, James Glasbey, Ian Ganly, Martin Batstone, Juan Rey Biel, Paul C Nankivell, Christian Simon, Omar Omar, Joana FF Simoes, Dmitri Nepogodiev, Aneel Bhangu, Tom Pinkney, Laura McGill, Rita Perry, Terry Hughes, Richard Jackson

***CovidSurg Operations Committee***

Kwabena Siaw-Acheampong, Ruth A Benson, Edward Bywater, Daoud Chaudhry, Brett E Dawson, Jonathan P Evans, James C Glasbey, Rohan R Gujjuri, Emily Heritage, Conor S Jones, Sivesh K Kamarajah, Chetan Khatri, Rachel A Khaw, James M Keatley, Andrew Knight, Samuel Lawday, Elizabeth Li, Harvinder S Mann, Ella J Marson, Kenneth A McLean, Siobhan C Mckay, Emily C Mills, Dmitri Nepogodiev, Gianluca Pellino, Maria Picciochi, Elliott H Taylor, Abhinav Tiwari, Joana FF Simoes, Isobel M Trout, Mary L Venn, Richard JW Wilkin, Aneel Bhangu.

***International Cancer Leads (*denotes specialty Principal Investigator)***

James C Glasbey (*Chair*); Head and neck: Richard Shaw*, Andrew G Schache, Stuart C Winter, Michael W S Ho, Paul Nankivell, Juan Rey Biel, Martin Batstone, Ian Ganly, Christian Simon.

***Dissemination Committee***

Joana FF Simoes (*Chair*); Tom EF Abbott, Michel Adamina, Adesoji O Ademuyiwa, Arnav Agarwal, Ehab Alameer, Derek Alderson, Felix Alakaloko, Markus Albertsmeiers, Osaid Alser, Muhammad Alshaar, Sattar Alshryda, Alexis P Arnaud, Knut Magne Augestad, Faris Ayasra, José Azevedo, Brittany K Bankhead-Kendall, Emma Barlow, Ruth A Benson, Ruth Blanco-Colino, Amanpreet Brar, Ana Minaya-Bravo, Kerry A Breen, Chris Bretherton, Igor Lima Buarque, Joshua Burke, Edward J Caruana, Mohammad Chaar, Sohini Chakrabortee, Peter Christensen, Daniel Cox, Moises Cukier, Miguel F Cunha, Giana H Davidson, Anant Desai, Salomone Di Saverio, Thomas M Drake, John G Edwards, Muhammed Elhadi, Sameh Emile, Shebani Farik, Marco Fiore, J Edward Fitzgerald, Samuel Ford, Tatiana Garmanova, Gaetano Gallo, Dhruv Ghosh, Gustavo Mendonça Ataíde Gomes, Gustavo Grecinos, Ewen A Griffiths, Madalegna Gründl, Constantine Halkias, Ewen M Harrison, Intisar Hisham, Peter J Hutchinson, Shelley Hwang, Arda Isik, Michael D Jenkinson, Pascal Jonker, Haytham MA Kaafarani, Angelos Kolias, Schelto Kruijff, Ismail Lawani, Hans Lederhuber, Sezai Leventoglu, Andrey Litvin, Andrew Loehrer, Markus W Löffler, Maria Aguilera Lorena, Maria Marta Madolo, Piotr Major, Janet Martin, Hassan N Mashbari, Dennis Mazingi, Symeon Metallidis, Ana Minaya-Bravo, Helen M Mohan, Rachel Moore, David Moszkowicz, Susan Moug, Joshua S Ng-Kamstra, Mayaba Maimbo, Milagros Niquen, Faustin Ntirenganya, Maricarmen Olivos, Kacimi Oussama, Oumaima Outani, Marie Dione Parreno-Sacdalanm, Francesco Pata, Carlos Jose Perez Rivera, Thomas D Pinkney, Willemijn van der Plas, Peter Pockney, Ahmad Qureshi, Dejan Radenkovic, Antonio Ramos-De la Medina, Keith Roberts, April C Roslani, Martin Rutegård, Irène Santos, Sohei Satoi, Raza Sayyed, Andrew Schache, Andreas A Schnitzbauer, Justina O. Seyi-Olajide, Neil Sharma, Richard Shaw, Sebastian Shu, Kjetil Soreide, Antonino Spinelli, Grant D Stewart, Malin Sund, Sudha Sundar, Stephen Tabiri, Philip Townend, Georgios Tsoulfas, Gabrielle H van Ramshorst, Raghavan Vidya, Dale Vimalachandran, Oliver J Warren, Duane Wedderburn, Naomi Wright, EuroSurg, European Society of Coloproctology (ESCP), Global Initiative for Children’s Surgery (GICS), GlobalSurg, GlobalPaedSurg, ItSURG, PTSurg, SpainSurg, Italian Society of Colorectal Surgery (SICCR), Association of Surgeons in Training (ASiT), Irish Surgical Research Collaborative (ISRC), Transatlantic Australasian Retroperitoneal Sarcoma Working Group (TARPSWG), Italian Society of Surgical Oncology (SICO).

***Collaborating authors***

**Argentina**: Boccalatte LA (***Hospital Italiano de Buenos Aires***).

**Australia:** Batstone M, Hodge R (***Royal Brisbane and Women's Hospital***).

**Belgium**: Abeloos J, De Backer T, De Ceulaer J, Dick C, Diez-Fraile A, Lamoral P, Spaas C (***AZ Sint-Jan Brugge-Oostende AV***); Schrijvers DLAL *(****Hospital Network Antwerp***); Willemse EBM (***Jules Bordet Institute***); Faris C, Maariën S, Van Haesendonck G, Van Laer C (***University Hospital Antwerp****);* Deron P (***University Hospital Gent***).

**Brazil**: Abdallah EA, Carvalho GB, Kowalski L, Vartanian J (***A.C. Camargo Cancer Center***); Gatti AP, Nardi CN, Oliva RNL (***Hospital Geral de Pirajussara***); Salem MC (***Irmandade da Santa Casa de Misericórdia de Porto Alegre***).

**Canada**: Cheng D, MacNeil D, Martin J, Mayer R, (***London Health Sciences Centre and St Josephs Health Care London***); Groot G (***Saskatoon City Hospital/Royal University Hospital/St. Paul's Hospital***).

**Colombia**: Acosta L, Mejia M, Perez CJ (***Fundacion Cardioinfantil-IC***).

**Croatia**: Lorencin M, Luksic I, Mamic M (***University Hospital Dubrava***).

**Egypt**: Ashoush FM (***Alexandria Main University Hospital***); Osman NA (***Alexandria Medical Research Institute***); Safwat Shahine M (***Assiut University Hospital***); Eldaly A (***El-Menshawy Hospital***); Elfiky MMA (***Kasr Alainy Hospital***); Amin A (***National Cancer Institute***); Elmorsi R, Refky B (***Oncology Center Mansoura University***); Essa MM (***Tanta University Hospital***).

**Ethiopia**: Mengistu G Mengesha (***Hawassa University Comprehensive Specialized Hospital***).

**France**: Dakpé s (***CHU Amiens***); Boucher S (***CHU Angers***); Ballouhey Q, Laloze J, Usseglio J (***CHU Limoges***); Hoffmann C (***Curie Institute***); Gregoire V (***Léon Bérard Cancer Center***); Lallemant B (***University Hospital of Nîmes***).

**Germany**: Blaurock M (***Greifswald University Medicine***); Reim D (***Klinikum Rechts der Isar TUM School of Medicine***); Boehm A (***Klinikum St. Georg, ENT Dept.***); Guntinas-Lichius O (***Universitätsklinikum Jena***); Hölzle F, Modabber A, Winnand P (***University Hospital Aachen***); Kleeff J, Lorenz K, Ronellenfitsch U, Schneider R (***University Hospital Halle, Saale***); Betz CS, Böttcher A, Busch C, Möckelmann N (***University Medical Center Hamburg-Eppendorf***); Inhestern JM (***Oberhavelkliniken Hennigsdorf***) Greve J, Hoffmann TK, Laban S, Vahl JM (***University Hospital Ulm***).

**Ghana**: Agyeman-Prempeh A, Aning D, Barnor I, Darko-Asante R, Dzogbefia M, Gaveh V, Gyimah D, Issahalq MD, Konney A, Poku M (***Komfo-Anokye Teaching Hospital***); Adjeso T, Akornor ET, Amankwaa WO, Antwi DA, Apppiah-Thompson P, Damah M, Kumi EO, Manan L, Murphy JP, Osei L, Setuagbe J (***Tamale Teaching Hospital***).

**Greece**: Arkadopoulos N, Danias N, Economopoulou P, Frountzas M, Kokoropoulos P, Larentzakis A, Michalopoulos NV, Nastos K, Parasyris S, Pikoulis E, Selmani J, Sidiropoulos TA, Vassiliu P (***Attikon University General Hospital***); Kalfountzos CE (***General Hospital of Larissa "Koutlimpaneio and Triantafylleio"***); Chatziioannou I, Corais C, Gkrinia E, Ntziovara A, Saratziotis A (***General University Hospital of Larissa***); Antoniadis K, Orestis O, Tatsis D (***George Papanikolaou General Hospital of Thessaloniki***); Baili E, Charalabopoulos A, Liakakos T, Schizas D, Spartalis E, Syllaios A, Zografos C (***Laiko University Hospital***).

**Guatemala**: Aguilera-Arévalo M (***Hospital General San Juan De Dios***).

**India**: Misra S, Pareek P, Vishnoi J (***All India Institute of Medical Sciences, AIIMS, Jodhpur***); Chappity P, Kar M, Muduly DK, Sultania M (***All India Institute Of Medical Sciences, Bhubhaneshwar***); Agarwal S, Garg PK, Maharaj DD, Majumdar KS, Mishra N, Poonia D, Seenivasagam RK, Singh MP, Tiwari AR (***All India Institute Of Medical Sciences, Rishikesh***); Penumadu P (***Jawaharlal Institute of Postgraduate Medical Education and Research***); Rajan S (***King George's Medical University, Lucknow, UP***); Kumar S (***Max Superspeciality Hospital***); Raychowdhury R (***Ramakrishna Mission Seva Pratishthan***); Ghodke R (***Sanjay Clinic & Apollo Hospital***); Raychowdhury R (***Vivekananda Institute of Medical Sciences***).

**Ireland**: Barry C, Callanan D, Dias A, Haung L, Ionescu A, Sheahan P (***South Infirmary Victoria University Hospital***); Lennon P, Fitzgerald C (***St. James's Hospital***).

**Israel:** Mizrachi A (***Rabin Medical Center***).

**Italy**: Deganello A (***ASST Spedali Civili, Ospedale di Brescia***); Pellini R, Pichi B (***Regina Elena National Cancer Institute***); Lemma F (***Azienda Ospedaliera di Padova***); Marino MV (***Azienda Ospedaliera, Ospedali Riuniti Villa Sofia, Cervello***); Bergonzani M, Varazzani A (***Azienda Ospedaliero-Universitaria di Parma***); Bussu F, Perra T, Piras A, Porcu A, Rizzo D (***Cliniche San Pietro, A.O.U. Sassari***); Campisi G, Cordova A, Franza M, Rinaldi G, Toia F (***Department of Surgical, Oncological and Oral Sciences, University of Palermo***); Gianni A (***Fondazione IRCCS Ca' Granda - ospedale Maggiore Policlinico***); Giannini L (***Fondazione IRCCS Istituto Nazionale dei Tumori, Milano***); Gordini L (***Fondazione Policlinico Universitario Agostino Gemelli***); Baldini E, Conti L (***G. Da Saliceto***); De Virgilio A, Ferreli F, Gaino F, Mercante G, Spriano G (***Humanitas University; IRCCS Humanitas Research Hospital***); Ansarin M, Chu F, De Berardinis R, Ietrobon G, Tagliabue M (***Istituto Europeo di Oncologia - IRCCS -Milano***); Ionna F (***Istituto Nazionale Tumori Fondazione, Pascale - IRCCS***); Baietti A, Maremonti P, Neri F, Prucher G, Ricci S (***Ospedale Maggiore/Bellaria Carlo Alberto Pizzardi AUSL Bologna***); Casaril A, Nama M (***Ospedale Pederzoli***); Cotoia A, Lizzi V, Vovola F (***Ospedali*** ***Riuniti Azienda Ospedaliera Universitaria Foggia***); Bruzzaniti P, Familiari P, Lapolla P, Marruzzo G, Mingoli A, Ribuffo D (***Policlinico Umberto I***); Cipriani R, Contedini F, Lauretta M, Marchetti C, Melotti M, Pignatti M, Pinto V, Pizzigallo A, Ricotta F, Tarsitano A (***Sant'Orsola Hospital, Alma Mater Studiorum University of Bologna, Italy***); Catarzi L, Consorti G (***University Hospital Umberto, Ancona***).

***Libya***: Abdulwahed EA, Alshareea EA, Biala MI, Ghmagh RJ (***Tripoli Central Hospital***).

**Malaysia**: Ibrahim AF (***Sarawak General Hospital***); Liew YT (***University Malaya Medical Centre***).

**Mexico**: Alvarez MR, Arrangoiz R, Cordera F, Gómez-Pedraza A (**ABC Medical Center**); Soulé-Martínez CE (***Hospital Central Norte Pemex***); Becerril OS (***Hospital Juárez de México***); Becerra GFC (***Hospital San Angel Inn Patriotismo***).

**Morocco**: Arkha Y, Bechri H, El Ouahabi A, Oudrhiri M (***Hôpital des Spécialités - ONO***); Benkabbou A, Majbar M, Mohsine R, Souadka A (***Universite Mohammed V Institut National d'Oncologie Rabat***).

**Nepal**: Lageju N (***Nepal Police Hospital***).

**Netherlands**: Schreuder WH (***Antoni van Leeuwenhoek Ziekenhuis***); Hardillo J (***Erasmus Medisch Centrum***); de Bree R (***University Medical Center Utrecht***); Schweitzer D (***Zuyderland Medical Center***).

**Nigeria**: Adeyeye AA, Enoch EE (***Afe Babalola University Multi-System Hospital***); Sholadoye TSTT (***Ahmadu Bello University Teaching Hospital***); Wuraola F (***Obafemi Awolowo University Teaching Hospitals Complex***); Oyelakin O (***University College Hospital, Ibadan***).

**Pakistan**: Khokhar MI (***Lahore General Hospital, PGMI, AMC***); Ayub B (***Patel Hospital***).

**Poland**: Walędziak M (**Military Institute Of Medicine**); Szewczyk M (***The Gerater Poland Cancer Center***).

**Portugal**: Faria C (***Centro Hospitalar Sao Joao***); Cardoso P (***Hospital Faro, Centro Hospitalar Universitario do Algarve***); Castro Silva J (***IPO Porto***).

**Saudi Arabia**: AlKharashi E (***Prince sultan military medical city***).

**Serbia**: Jelovac D, Petrovic M, Sumrak S (***Clinic for Maxillofacial Surgery, School of Dental Medicine, University of Belgrade***); Asceric RR, Bojicic JM, Kovacevic BM, Krdzic ID, Milentijevic MA, Milutinovic VZ, Stefanovic ZB (***Zvezdara University Medical Center***).

**Spain**: Villacampa JM (***Fundación Jimenez Diaz University Hospital***); Jiménez Carneros V, Salazar Carrasco A, Carabias Hernandez A, Alonso Lamberti L, León Ledesma R, Jiménez Miramón FJ, Jover Navalón JM, Garcia Quijada J, Ramos Rodriguez J, Valle Rubio A (***Getafe University Hospital***); Vilaseca I (***Hospital Clínic of Barcelona***); Escartin J (***Hospital Royo Villanova***); Estaire-Gomez M, Padilla Valverde D (***Hospital General Universitario de Ciudad Real***); Tousidonis M (***Hospital General Universitario Gregorio Marañón***); Lopez F (***Hospital Universitario Central de Asturias, HUCA***); Deandrés-Olabarria U, Durán-Ballesteros M, Fernández-Pablos F, Ibáñez-Aguirre F, Sanz-Larrainzar A, Ugarte-Sierra B (***Hospital Universitario de Galdakao***); Di Martino M, Prada J (***Hospital Universitario de la Princesa***); Jariod-Ferrer UM (***Hospital Universitario Miguel Servet***); Landaluce Olavarria A (***Hospital Urduliz***); Rey-Biel J (***Rey Juan Carlos University Hospital***); Díaz de Cerio P (**Saint Peter Hospital**); Sánchez Barrueco A (***Villalba University General Hospital***).

**Sweden**: Lindqvist EK (***Karolinska University Hospital***); Sund M (***Umea University Hospital***).

**Switzerland**: Piantanida R (***Ente Ospedaliero Cantonale***); Giger R, Hool S, Müller SA (***Inselspital, Bern University Hospital, University of Bern***); Stoeckli SJ (***Kantonsspital St. Gallen***); Simon C (***Lausanne University Hospital CHUV***).

**Syrian Arab Republic**: Toutounji T (***Aleppo University Hospital***);Al assaf A (***Al-Mouwasat University Hospital***); Hammed AM, Hammed SM, Mahfoud M (***Tishreen University Hospital***).

**Turkey**: Arikan A (***Acibadem Maslak Hospital***); Yalkin Ö (***Ali Osman Sönmez Oncology Hospital***); İflazoğlu N (***Bursa City Hospital***); Isik A, Leventoglu S (***Erzincan University Hospital***); Aydemir L, Basaran B, Sen C, Comert -Ulusan M (***Istanbul University Faculty of Medicine***); Basaran B (***Karaman Training and Research Hospital***); Saracoglu KT (***Kartal Dr. Lutfi Kirdar Training and Research Hospital***); Saracoglu A (***Marmara University, School of Medicine***); Mantoglu B (***Sakarya Faculty Of Medicine***); Kucuk G (***Samsun Training and Research Hospital***); Aygun N, Baran E, Tanal M, Eray Tufan A, Uludag M, Gürkan Yetkin S, Yigit B (***Sisli Hamidiye Etfal Training and Research Hospital***); Calik B, Demirli Atici S, Kaya T (***University of Health Sciences Tepecik Training and Research Hospital***).

**Uganda**: Sikakulya FK (***Kampala International University Teaching Hospital***).

**United Arab Emirates**: Abdel-Galil KMAH (***Tawam Johns Hopkins Hospital***).

**United Kingdom**: Lowe T (***Aberdeen Royal Infirmary***); Durrani AJ, Habeeb A, Irune E, Luke L, Masterson L, Murphy SH, Segaren N, Walker C, Waseem S (***Addenbrooke's Hospital***); Jones TM, Loh C, Pringle S, Schache AG, Shaw RJ (***Aintree University Hospital***); Stenhouse J (***Altnagelvin Area Hospital***); Armstrong M (***Borders General Hospital***); Sood S, Sutton D (***Bradford Royal Infirmary***); Thomas S (***Bristol Royal Infirmary***); Clarke P (***Charing Cross Hospital***); Winter SC (**Churchill Hospital**); Hislop S (***Crosshouse University Hospital***); Counter PR (***Cumberland Infirmary***); Ghazali N (***East Lancashire Hospitals NHS Trust***); Lloyd C (***Glan Clwyd Hospital***); Prabhu V (***Glangwili General Hospital***); Godden D, Whitley S (***Gloucestershire Royal Hospital***); Butler C, Nash R (***Great Ormond Street Hospital for Children***); El-Boghdadly K, Fry A, Niziol R (***Guy's Hospital***); De M, Gill CK (***Heartlands Hospital***); Crank S (***Hull University Teaching Hospitals NHS Trust***); Mace AD (***Imperial College NHS Trust***); Ho M (***Leeds General Infirmary***); Mair M (***Leicester Royal Infirmary***); Kothari P (***Luton and Dunstable University Hospital***); Homer J, Sainuddin S (***Manchester Royal Infirmary***); Egan RJ, Kittur M (***Morriston Hospital Swansea***); Burgess C (***Musgrove Park Hospital)***; O'Hara J (***Newcastle Upon Tyne Hospitals NHS Foundation Trust***); Manickavasagam J, McDonald C (**Ninewells Hospital**); Burrows S (***Norfolk and Norwich University Hospital***); Java KR, Katre C (***North Manchester General Hospital***); Ahmed A, Siddique H (***Northwick Park Hospital***); King E, Ramchandani P (***Poole Hospital***); Naredla PR (***Princess Royal Hospital***) Brennan P, Ringrose T, Schmidt F (***Queen Alexandra Hospital***); Mak JKC, Nankivell P, Parmar S, Sharma N (***Queen Elizabeth Hospital Birmingham***); Douglas C, McCaul J, McCaul J (***Queen Elizabeth University Hospital***); Dhanda J (***Queen Victoria Hospital***); Ghazali N, Kyzas P, Vassiliou L (***Royal Blackburn Hospital***); Kumar A (***Royal Derby Hospital***); Husband A (***Royal Devon and Exeter Hospital***); Hulbert J (***Royal Glamorgan Hospital***); Ingrams D, Parkin R (***Royal Gwent Hospital***); Varley I (***Royal Hallamshire Hospital***); Gahir D, George A, Zakai D (***Royal Stoke University Hospital***); Bater M (***Royal Surrey County Hospital***); Surwald C (***Royal Sussex County Hospital***); Devlin B, Leonard CG (***Royal Victoria Hospital***); Pigadas N, Snee D (***Royal Wolverhampton NHS Trust***); Singh RP (***Southampton General Hospital***); Hyde NC (***St George's Hospital***); Paley M (***St John's hospital, Livingston***); Cocks H, Wilson A (***Sunderland Royal Hospital***); Choi D (***The National Hospital for Neurology and Neurosurgery***); Kerawala CJ, Riva F (***The Royal Marsden NHS Foundation Trust***); Dickason A (***Torbay and South Devon NHS Trust***); Semple CJ (***Ulster Hospital Dundonald***); Schilling C (***University College London Hospital at Westmoreland Street***); Naredla PR, Walton G (***University Hospitals Coventry and Warwickshire NHS Trusts***); Rees-Stoner O (***University Hospitals Plymouth***); Scott N (***University Hospital of Wales***); Nixon IJ (***Western General Hospital***); Tighe D (***William Harvey Hospital***); Mattine S (***Worcestershire Royal Hospital***); Chu MMH, Pothula V (***Wrightington, Wigan & Leigh NHS Foundation Trust***).

**United States**: Lee W (***Duke University Medical Center***); Brown L, Ganly I (***Memorial Sloan Kettering Cancer Center***); Alpert N, Illezeau CN , Miles B, Rapp J, Taioli E (***Mount Sinai Hospital***); Azam MT, Choudhry AJ, Marx W (***SUNY Upstate University Hospital***); Stein J (***The University Hospital***); Ying Y (***University of Alabama Birmingham***); Gross ND (***University of Texas MD Anderson Cancer Center***); Almasri M, Joshi R, Kulkarni G, Marwan H, Mehdi M (***University of Texas Medical Branch***); Sumer B (***UT Southwestern Medical Center***).

**Acknowledgements**

COVIDSurg Collaborative

Birmingham Centre for Observational and Prospective Studies (BiCOPS)

British Association of Head & Neck Oncologists (BAHNO)
